# Care of Children

**Published:** 1879-08

**Authors:** 


### CARE OF CHILDREN.

This most important subject I have reserved for the last in this article, with a view of making it more impressive.

Childhood is the season in which, by proper care and attention, we may transform a constitution, delicate and frail by inheritance, into one of robust health.

The responsibility for half the bodily suffering endured in maturer years can be traced back to inattention or want of judgment on the part of parents toward their offspring during infancy and childhood. The first and most important question to be settled, on the arrival of a "little stranger" on this mundane sphere, is, how shall he be nourished? On the decision of this question the future health and happiness of the infant may chiefly depend.

It is a physiological fact, generally admitted, that perfect health of body and tranquillity of mind are essential to a healthy secretion of milk, and that the fluid becomes vitiated by bodily ailments and mental troubles. To nurse a child under these circumstances is to force upon it an inheritance of disease. There are few women so stupid as not to mingle to some extent in the whirl and excitement of this age; consequently, there are but few women who are fit to be mothers in all respects. I have been a close observer of these facts for many years. I believe I can go into a family of ever so many children, that I have never seen before, and if there is but one child in the number who has been "raised on the bottle'—that is, on cow's milk—I can instantly point to that child. He will be, almost invariably, the healthiest, happiest, and brightest of the number. Except, then, in rare cases, I am in favor of raising children on cow's milk. Reader, don't raise your hands in holy horror and say it is opposed to nature! When healthy mothers lead natural and tranquil lives, I will concede the point; till then I have the best of the argument.

